# DL-β-Aminobutyric Acid-Induced Resistance in Soybean against *Aphis glycines* Matsumura (Hemiptera: Aphididae)

**DOI:** 10.1371/journal.pone.0085142

**Published:** 2014-01-15

**Authors:** Yunpeng Zhong, Biao Wang, Junhui Yan, Linjing Cheng, Luming Yao, Liang Xiao, Tianlong Wu

**Affiliations:** Key Laboratory of Urban Agriculture (South) Ministry of Agriculture, School of Agriculture and Biology, Shanghai Jiao Tong University, Shanghai, China; Federal University of Viçosa, Brazil

## Abstract

Priming can improve plant innate capability to deal with the stresses caused by both biotic and abiotic factors. In this study, the effect of DL-β-amino-n-butyric acid (BABA) against *Aphis glycines* Matsumura, the soybean aphid (SA) was evaluated. We found that 25 mM BABA as a root drench had minimal adverse impact on plant growth and also efficiently protected soybean from SA infestation. In both choice and non-choice tests, SA number was significantly decreased to a low level in soybean seedlings drenched with 25 mM BABA compared to the control counterparts. BABA treatment resulted in a significant increase in the activities of several defense enzymes, such as phenylalanine ammonia-lyase (PAL), peroxidase (POX), polyphenol oxidase (PPO), chitinase (CHI), and β-1, 3-glucanase (GLU) in soybean seedlings attacked by aphid. Meanwhile, the induction of 15 defense-related genes by aphid, such as *AOS*, *CHS*, *MMP2*, *NPR1-1*, *NPR1-2*, and *PR* genes, were significantly augmented in BABA-treated soybean seedlings. Our study suggest that BABA application is a promising way to enhance soybean resistance against SA.

## Introduction

Plants are able to protect themselves against attack by pathogens and pests through constitutive and inducible defense mechanisms, such as rapid synthesis of toxic metabolites and defensive proteins. Following specific stimulation, plant resistance will be elevated and plants acquire enhanced protection against future pathogen attack, a phenomenon known as induced resistance. Such induced defenses are generally recognized to impose a resource cost on the plant, manifested as reduced growth and reproductive fitness [1]. BABA, a non-protein amino acid, can induce plants into a sensitization state in which defenses are not expressed, but in which plants are able to respond more rapidly and/or more strongly to attack than other plants that have not experienced previous stress. The BABA-mediated resistance augment in plants is called primed state. Priming switches plants into an alarmed state of defense and consequently upgrades the plant defensive capability [2], [3]. Priming offers effective and economic protection against plant diseases, especially in the area with relatively high disease pressure [4].

*Aphis glycines* Matsumura (Hemiptera: Aphididae), known as the soybean aphid (SA), is a predominant insect pest of soybean [*Glycine max* (L.) Merr.] in some Asian countries and North America. It is the primary aphid species known to colonize soybean in North America [5]. The soybean aphid is highlighted as one of the top insect pest constraints to soybean production worldwide [6]. The economic impact of the soybean aphid on soybean production has been estimated to range from US $3.6 to $4.9 billion annually in North America [7].

The production loss is partially due to direct damage on plants caused by SA colonization, such as plant stunting, leaf distortion, and reduced pod set [8]; the indirectly reasons were the soybean virus transmission and the buildup of black sooty mold on honeydew produced by SA [9]. Also the production cost of soybean is increased because of extensive use of insecticide. However, the interaction among different components of integrated management for soybean aphid, such as host resistance, biological control and chemical prevention is still unknown [10].

DL-β-amino-n-butyric acid (BABA) has been known to confer protection against a broad spectrum of biotic and abiotic stresses, such as *Peronospora parasitica* in *Arabidopsis thaliana* L. [11], *Sclerotinia sclerotiorum* in *Cynara cardunculus* L. [12], *Bremia lactucae* in *Lactuca sativa* L. [13], [14], *Pseudomonas syringae* pv. tomato in *Arabidopsis thaliana* L. [15], *Meloidogyne javanica* in *Cucumis sativus* L. [16], *Myzus persicae* in *Sinapsis alba* L. [17], and *Acyrthosiphon pisum* in *Vicia faba* L. var. minor [18]. However, the function of BABA in soybean resistance against SA is largely unknown. In order to protect soybean from SA attack, we investigated the effect of BABA in inducing priming in soybean and provided evidences to the possible mechanisms.

## Materials and Methods

### Plant material and BABA treatment

To screen an optimal concentration of BABA, seeds of soybean cultivar Dongnong 47 (SA-susceptible) were sterilized by 0.1% calcium hypochlorite, and then sowed in the humid sterilizing perlite. After five days, the seedlings were transferred to 10 cm-deep×8 cm-diameter plastic cups filled with 60 g perlite in an environment-controlled greenhouse at 26°C with a 14 h photoperiod and 70% relative humidity (RH). Seedlings were irrigated with 25 ml Hogland solution for each cup when needed and a week later, each cup was irrigated with 25 ml BABA-water solution with different concentrations (0, 10 mM, 25 mM, 50 mM, 75 mM, and 100 mM). Each treatment was repeated for 20 times. After 10 days, the seedlings were sampled for analyzing the growth indexes including plant height, fresh weight, dry weight, root length and root vitality.

To analyze the physiological responses and the genes expression levels of soybean after SA attack, seeds (Dongnong 47) were sowed in 8 cm-deep×10 cm-diameter plastic pots filled with 150 g uniform matrix (100 g matrix with 50 ml water) in the same greenhouse. After 12 days, 25 ml BABA-water solution was applied as soil drench. Water was withheld for 3 days. Soybean seedlings were maintained in the greenhouse for the duration of the study.

DL-β-amino-n-butyric acid (BABA; purity >97%, A44207) was obtained from Sigma-Aldrich Ltd, Shanghai, China.

### Determination of DI (damage index) of BABA to soybean seedlings

In the BABA concentration optimization experiment, we found BABA inhibited the growth of soybean seedlings in a concentration-dependent manner and distinguished the DI according to the symptoms of the first pair of primary leaves and the first trifoliolate leaves. The grade scale was as follows: I, healthy with no symptoms; II, primary leaves yellowing with less than 50% proportion and the first trifoliolate leaf was normal; III, primary leaves yellowing with more than 50% proportion and the size of first trifoliolate leaves was smaller; IV, primary leaves drying and the size of first trifoliolate leaves was smaller; V, both of primary leaves and the first trifoliolate leaves drying.

### Aphid culture, inoculation, non-choice and choice tests

Soybean aphids were obtained from a laboratory-maintained colony. The original soybean aphid colony was acquired from the experimental fields of School of Agriculture and Biology, Shanghai Jiaotong University in August 2006. The colony was preserved on Dongnong47 soybean seedlings (V1–V4 stages). Every two months, new plants were provided to the colony and aphids were transferred by placing infested leaves on new plant leaves. SA used in all experiments were synchronized as described previously [19]. Several viviparous apterae were placed on detached leaves of Dongnong 47 in petri dishes containing moist filter paper for 24 h. All of the viviparous apterae were removed after 24 h leaving only 1-day-old nymphs. For easier handling and improved survivability of soybean aphids, third instar nymphs were collected from the dishes to infest plants.

Non-choice and choice tests were performed according to previously published methods [20], [21]. After 3 days of BABA-drenched, seedlings were inoculated with 6 apterous aphids on the upper side of the trifoliolate leaf of each plant with a moist brush slightly. Aphids were confined (non-choice) or not confined (choice) to individual plants using a tubular polycarbonate plastic cages (15 cm diameter×50 cm height) with organdy fabric secured by rubber bands at the top. Each test was repeated for 15 times. In the second choice test, 20 aphids were placed on a sterile filter paper surrounded with four seedlings (two drenched with 25 ml 25 mM BABA and two with water, respectively) in a big pot (28 cm-deep×32 cm-diameter, n = 16) and the SA number of the two groups were counted 7 days later. All the plants were in a random arrangement.

### Determination of SA number, weight and growth parameters

After BABA priming for 3 days, the number of SA were counted at the designed time points post inoculation (n = 15). To determine weight for single aphid, the total weight of 30 apterous aphids with different ages was measured for 6 times and the mean value was calculated as the weight of single aphid. Seven days post inoculation, MRGR (Mean Relative Growth Rate) and MRRR (Mean Relative Reproduction Rate) were calculated using the following formula [22]:

MRGR =  [log_e_ (final weight)-log_e_ (initial weight)]/7 days g g^−1^ d^−1^

MRRR =  (Final number−Initial number)/7 days No. d^−1^

### Determination of defensive enzyme activities

Three days later after BABA priming, the trifoliolate leaves of seedlings were sampled at 1, 3, 5 and 7 days after SA inoculation, and then were stored at −80°C for protein and defense enzymes activity assays, all the data were repeated for three times.

Chitinase and β-1, 3-glucanase activity as markers for SAR (Systemic Acquired Resistance) [23] were determined. Three enzymes related to phenylalanine and phenolic metabolism were also assayed for the possible mechanisms of BABA priming.

To determine activities of defense enzymes, 1 g leaf tissue was homogenized with 5 ml extraction buffer [0.05 M phosphate buffer (pH 6.8) for phenylalanine ammonia-lyase (PAL), peroxidase (POX) and polyphenoloxidase (PPO); 0.1 M acetic acid buffer (pH 5.0) for chitinase (CHI) and β-1,3-glucanase (GLU)] and centrifuged at 12,000×g for 5 min at 4°C. The supernatant was collected and stored at −80°C until analysis.

PAL (EC 4.3.1.5) (phenylalanine ammonia-lyase) activity was determined as described by Koukol and Conn [24] with minor modifications. An aliquot (500 µl) of the extract was incubated with 1 ml 0.02 M l-phenylalanine and 2 ml 0.2 M boric acid buffer (pH 8.8) at 30°C for 1 h, after which absorbance at 290 nm was measured. PAL activity was expressed as Ug^−1^ protein, where U = A290 h^−1^.

PPO (EC 1.10.3.2) (polyphenol oxidase) activity was determined as described by Liu [25] with some modifications. An aliquot (200 µl) of the extract was reacted with 2 ml buffered substrate (0.05 M phosphate buffer, pH 6.8) and 1 ml 0.1 M catechol, and the change in absorbance at 420 nm was recorded over 3 min. Specific activity is expressed as Ug^−1^ protein, where one unit is defined as an increase of 1 at OD 398 per min.

POX (EC 1.11.1.7) (Peroxidase) activity was determined by measuring the increase in absorbance at λ_max_ of 475 nm due to oxidation of guaiacol [26]. The 2 ml reaction mixture used for this case consisted of 25 mM of phosphate buffer with pH of 7.0, 0.05% guaiacol (w/v), 1.0 mM of H_2_O_2_, 0.1 mM of EDTA, and 0.2 ml of the root extract. Activity was expressed as the change in absorbance of the reaction mixture at 475 nm per mg of total protein per min.

CHI (EC 3.2.1.14) (chitinase) activity was measured according to Boller [27] with some modifications. An aliquot (500 µl) of the extract was mixed with 0.5 ml colloidal chitin and incubated at 40°C for 1 h. Then, 0.1 ml 20 g L^−1^ desalted snailase was added, and the mixture was incubated at 37°C for 1 h. The reaction was stopped by addition of 0.3 ml of 0.6 M potassium tetraborate and boiling for 5 min. After cooling, 2 ml of 100 g L^−1^ 4- (dimethylamino) benzaldehyde reagent diluted with glacial acetic acid (1∶5 v/v) was added and the mixture was incubated at 37°C for 20 min, and then absorbance was measured at 585 nm. CHI activity is expressed as Ug^−1^ protein, where one unit is defined as 10^−6^ mol N-acetyl-D-glucosamine produced per hour under these assay conditions.

GLU (EC 3.2.1.39) (β-1, 3-glucanase) activity was assayed as described by Siefert [28] with some modifications. Crude extract (100 µl) was mixed with 50 µl 0.4% laminarin, and the mixture was incubated for 1 h with shaking at 37°C. The reaction was stopped by addition of 200 µl 3, 5-dinitrosalicylic acid and boiling for 5 min. The mixture was then cooled to room temperature and determined the absorbance at 500 nm. Enzyme activity is expressed as Ug^−1^ protein, where a unit is defined as the formation of 1 nmol glucose equivalents released from laminarin per hour under these assay conditions.

Protein content was determined according to Bradford [29] with bovine serum albumin (Sigma-Aldrich, Shanghai, China) as the standard. All spectrophotometric analyses were conducted on the BioMate 3 S Spectrophotometer (Thermo Fisher scientific).

### Real-time quantitative RT-PCR

After BABA or water drenched for 3 days, primary leaves were inoculated with 6 apterous aphids as follows: to confine aphids movement, a 2.5 cm×2.5 cm piece of sticky plastic sheet with a 1.5 cm-diameter hole in the centre was sticked to the upper side of the leaflet of each plants and 6 apterous aphids were placed in each hole with a moist brush slightly, and then covered the hole with organdy fabric [30]. After 12, 24, 36 and 48 h, the attacked sites were cut by surgical scissors and aphids were removed quickly using a moist brush. Leaf samples at 0 h were not inoculated with aphids and also brushed when collected. Aphid- or mock-treated leaves for each of six plants were pooled together (three replications for each treatment), respectively, and immediately frozen in liquid nitrogen. All of the leaves were stored at −80°C before RNA isolation. All gene-specific primers were described in Table 1. Relative expression levels at each time point were calculated from cycle threshold (CT) values according to the ΔCT method (Applied Biosystems User Bulletin#2) using the soybean actin gene as a reference[31]–[43].

**Table 1:** Oligonucleotide primers used for quantitative real-time PCR.

Gene	Forward/reverse primers	Target sequence^a^	Tm(°C)^b^	Amplicon (bp)	Description	Reference
*Actin*	F: 5′-GAGCTATGAATTGCCTGATGG-3′	U60500	58	118	Soybean actin	[31]
	R: 5′-CGTTTCATGAATTCCAGTAGC-3′					
*PR1*	F: 5′-TGTGTTGTGTTTGTTAGGGTTAGTCA-3′	AF136636	61	137	PR1a precursor antimicrobial protein	[32]
	R: 5′-TGTTGGTGAGTCTTGAGCATACG-3′					
*PR2*	F: 5′-GTCTCCTTCGGTGGTAGTG-3′	M37753	57	104	Beta-1,3-Endoglucanase	[33]
	R: 5′-ACCCTCCTCCTGCTTTCTC-3′					
*PR3*	F: 5′-GCACTTGGTCTGGATTTG-3′	AF202731	53	115	Chitinase class I	[34]
	R: 5′-GGCTTGATGGCTTGTTTC-3′					
*PR12*	F: 5′-CATGGACAAGGCACGATTTGG-3′	BU964598	62	108	Defensin precursor	[35]
	R: 5′-AACCGATGGCTCTTTGACTCAC-3′					
*AOS*	F: 5′-CCTCTGTCTCCGAGAAACC-3′	DQ288260	59	120	Allene oxide synthase	[36]
	R: 5′-CCTTCAAGGGACCGATCAC-3′					
*CHS*	F: 5′-AGGCTGCAACTAAGGCAATC-3′	X53958	57	103	Chalcone synthase	[37]
	R: 5′-TAATCAGCACCAGGCATGTC-3′					
*IPER*	F: 5′-CTCTCAGGTGCTCATACATTCG-3′	AF007211	62	90	Basic peroxidase	[38]
	R: 5′-TGGATCAGGTTTGCCAGTTC-3′					
*MMP2*	F: 5′-GCGGAAGAAACTGAGGGAGTATT-3′	AY057902	59	91	Matrix metalloproteinase 2	[39]
	R: 5′-CGTCTTTTGTTCTACACGATCCAT-3′					
*P21-like*	F: 5′-TTACATAAGGCGTGTGCACTTTG-3′	XM_003525364	56	73	similar to PR 5 response to soybean aphid	[30]
	R: 5′-CCATTTCATTTAGAATAGAAGTACACACATC-3′					
*PAL*	F: 5′-GTGCAAGGGCTGCTTATG-3′	X52953	57	107	Phenylalanine ammonia-lyase	[40]
	R: 5′-CCCAGTCCCTAATTCCTCTC-3′					
*PPO*	F: 5′-GGGTTGGTGCTGCTGATAAG-3′	EF158428	62	100	Polyphenol oxidase	[41]
	R: 5′-CGATCCGAGTTCGTGTGATG-3′					
*GmNPR1-1*	F: GGGGATGCCTGTATGTCTTC-3′	FJ418594	56	169	Orthologous to AtNPR1 participation in SAR	[42]
	R: CGCAGAAAGACCAGCAAACT-3′					
*GmNPR1-2*	F: 5′-GTTGACAGTGTGTGTGCCCA-3′	FJ418596	56	175	Orthologous to AtNPR1 participation in SAR	[42]
	R: 5′-AACAGTGAGGATTGGGATGACA-3′					
*GmSGT1*	F: 5′-TGAGGCTGTGGCTGATGCTA-3′	NM_001249656	56	127	Participation in SAR	[43]
	R: 5′-ACCTCCAGAGCAGCCTTTG-3′					
*GmRAR1*	F: 5′-TGCTCCGAAACCTAAGAAGATA-3′	FJ222386	56	166	Participation in SAR	[43]
	R: 5′-ATCACAGCACTTCCACCCTC-3′					

^a^ NCBI accession number of Glycine max gene.

^b^ Primer annealing temperature.

### Statistical analysis

Data were analyzed for significant differences by analysis of variance (ANOVA) using the statistical software SPSS 16.0 for Windows (SPSS Inc., Chicago, IL, USA). Significant effects were determined using Fisher's LSD test (P<0.05) or two-tailed *t* test (P<0.01).

## Results

### Effects of BABA treatment on growth of soybean seedlings

**Figure 1:**
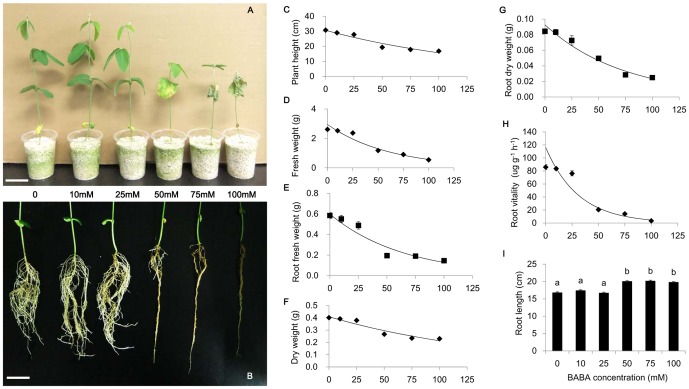
Effects of different concentrations of BABA treatment on the growth of soybean seedlings. (A) Symptoms of overground part. (B) Symptoms of root. (C) Plant height. (D) Fresh weight. (E) Root fresh weight. (F) Dry weight. (G) Root dry weight. (H) Root vitality. (I) Root length. Soybean seedlings were drenched with 25 ml BABA-water solution with different concentrations (0, 10 mM, 25 mM, 50 mM, 75 mM, and 100 mM) for 10 days. Bars indicate Mean±SE of 20 replicate samples (Root vitality was repeated for 3 times), and the lines are fitted concentration-response curves. Different letters indicate significant differences according to Fisher's LSD test (P<0.05, F = 45.72>F_0.05_ (5, 114) = 2.29). (Fig.1A, scale bar = 5 cm; Fig.1B, scale bar = 4 cm.).

To minimize the adverse impact of BABA when using as an agent against soybean aphid, the biological effects of this chemical were evaluated on the host plant growth. Soybean seedlings were grown under a normal condition with the addition of different amount of BABA. Several indexes were used to examine plant growth, which included fresh weight, dry weight, plant height, root length, and root vitality (Figure 1; Table S1), and the results of regression analysis were showed in Table 2. Low concentration of BABA supplements (≤25 mM) had a tiny influence on plant growth, because all analyzed growth indexes were comparable between treated and untreated seedlings (Figure 1A). However, high concentration (≥50 mM) BABA revealed an appreciable growth inhibitory effect, resulting in significant reduction of plant high, fresh weight, dry weight, and root vitality (with the exception of root length that was slightly increased). Additionally, high concentration of BABA retarded the development of leaf and greatly reduced the number of lateral root (Figure 1B). The roots treated with high concentration of BABA displayed yellowing symptom, suggesting a serious reduction in root vitality (Table 1H). Owing to the reduction of lateral root number and root vitality by BABA treatment, roots absorption potential can be greatly attenuated. Therefore, inhibition of soybean development by BABA is likely through restraining absorption of water and nutritive elements.

**Table 2:** Results of regression analysis.

Indexes		SS	df	MS	F	P	R^2^	Equation
Fresh weight	Regression	2.034	1	2.034	125.198	0.000	0.969	y = 2.9501e^−0.016x^
	Residual	0.065	4	0.016				
	Total	2.099	5					
Root fresh weight	Regression	1.804	1	1.804	40.163	0.003	0.909	y = 0.5983e^−0.015x^
	Residual	0.18	4	0.045				
	Total	1.983	5					
Plant height	Regression	0.34	1	0.34	50.345	0.002	0.926	y = 30.673e^−0.007x^
	Residual	0.027	4	0.007				
	Total	0.367	5					
Dry weight	Regression	0.321	1	0.321	44.234	0.003	0.917	y = 0.4092e^−0.007x^
	Residual	0.029	4	0.007				
	Total	0.35	5					
Root dry weight	Regression	1.425	1	1.425	114.812	0.000	0.966	y = 0.0927e^−0.014x^
	Residual	0.05	4	0.012				
	Total	1.475	5					
Root vitality	Regression	7.851	1	7.851	74.268	0.001	0.949	y = 116.15e^−0.032x^
	Residual	0.423	4	0.106				
	Total	8.274	5					
DI	Regression	9.53	1	9.53	83.226	0.001	0.954	y = 0.0355x+1.2471
	Residual	0.458	4	0.115				
	Total	9.988	5					

The independent variable is X (BABA concentration).

### DI analysis of BABA treatment on soybean seedlings

**Figure 2:**
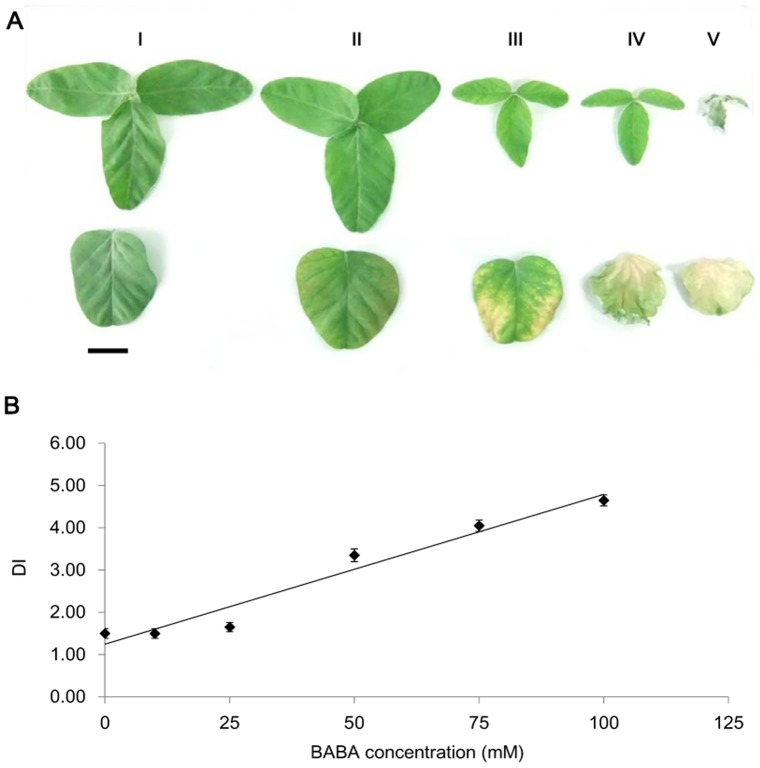
Damage index (DI) of soybean seedlings treated by BABA. (A) Grade scale of DI. (B) DI of seedlings after BABA-drenched for 10 days in the concentration optimization experiment. Bars indicate Mean±SE of 20 replicate samples, and the line is fitted concentration-response curves.

Besides the symptoms described above, BABA caused lesion on the treated soybean seedlings, as indicated by yellowing, wilting and even drying leaves. We classified the DI scales from 1 to 5 based on the wilting extent of the primary and the first trifoliolate leaves together with the size of yellowing spots. DI scales of seedlings drenched with different concentrations of BABA (from low to high) were indicated in Figure 2 and Table S2. The results of regression analysis were showed in Table 2. A dose-dependent lesion was found on BABA-treated seedlings; whereas there was no significant damage on the seedlings drenched with 25 mM or less BABA (P<0.05). Therefore, we concluded that 25 mM was an optimal BABA concentration as a root drench for soybean seedlings, which was adopted in the subsequent experiments unless otherwise indicated.

### Effect of BABA-induced resistance against SA

**Figure 3:**
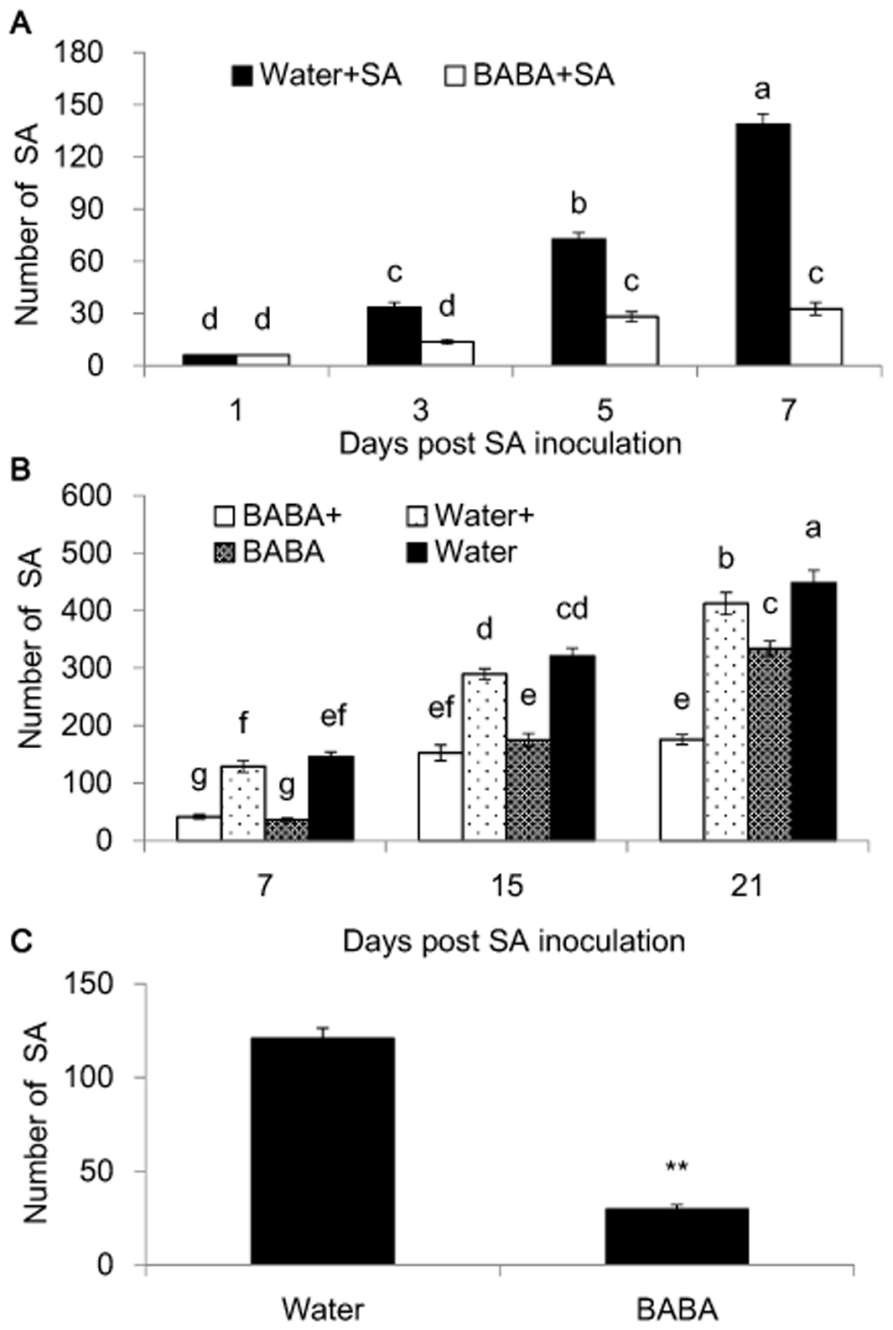
Numbers of soybean aphids on soybean seedlings drenched with BABA or water. (A) SA number of seedlings primed by BABA or water post SA inoculation in a short period (n = 15). (B) SA number of seedlings in choice and non-choice tests in a prolonged period (n = 15). BABA+ and Water+: non-chioce test; BABA and Water: choice test. (C) SA number of seedlings primed by BABA or water in the second choice test (n = 16). The seedlings were pre-treated with 25 ml 25 mM BABA or water for 3 days. Different letters indicate significant differences between treatments according to Fisher's LSD test (P<0.05). Bars indicate mean±SE of 15 or 16 replicate samples. Asterisks indicate statistically significant differences between treatments (**P<0.01; two-tailed *t* test).

SA number on the seedlings drenched with 25 mM BABA was decreased significantly at 3, 5, 7 days post SA inoculation (P<0.01). The BABA-mediated restriction for SA reproduction was observed at the early time point (3 days post inoculation). This persistent effect occurred during the whole test period and the SA number was reduced to 23.7% on the BABA-treated seedlings compared to the control (Figure 3A; Table S3). We further estimated the weight of single aphid (regardless of age) by weighing 30 aphids six times (Table 3). MRRR was markedly decreased from 19.00 to 3.86 due to BABA treatment; accordingly MRGR was estimated to be 0.24 and 0.45 for the BABA-treated and untreated seedlings (Table 4). The reason for estimating the MGRG and MRRR at 7 days post inoculation was the average population doubling time of 6.8 d±0.8 d (mean±SEM) according to the filed study [44].

**Table 3:** Average weight of single aphid (g).

Group	Total weight of 30 aphids	Weight of single aphid
1	0.0089	2.97E-04
2	0.0079	2.63E-04
3	0.0085	2.83E-04
4	0.0068	2.27E-04
5	0.0071	2.37E-04
6	0.0077	2.57E-04
Average weight of single aphid		2.61E-04
Standard Deviation		2.67E-05

**Table 4:** The effect of BABA as root drenched on MGRG and MRRR of SA at 7 days post inoculation.

Treatments	MRGR (g g^−1^ d^−1^) Mean Relative Growth Rate	MRRR (No. d^−1^) Mean Relative Reproduction Rate
Water+SA	0.45	19.00
BABA+SA	0.24	3.86

SA: soybean aphids; n = 15.

To further address the effect of BABA on restriction of SA, we designed non-choice and choice tests with a prolonged period. At 7 days and 15 days post SA inoculation, SA number on plants drenched with water in the non-choice (n = 15) and choice (n = 15) test had no significant difference (P<0.05) and similar results were also observed for the BABA-treated plants. However, the insect number in the choice test was higher than that in the non-choice test at 21 days post aphid inoculation (P<0.05) (Figure 3B; Table S3). Less aphids in both tests were found on BABA-treated plants compared to the mock controls at all time points. At 21 days, SA average number was 334 and 449 on BABA-treated and control soybean seedlings in the choice test, respectively. These results demonstrated that priming by 25 mM BABA inhibited the weight and production rate of SA.

In the second choice test, the SA number was 30 and 121 on BABA- and water-treated plants, respectively, and the difference reached a significant level (Figure 3C, P<0.01; Table S3). These results suggested that BABA could alter the behavior orientation of SA and inhibit its reproduction rate.

### BABA-mediated activation of defense enzymes are correlated with the soybean resistance against SA

To understand the mechanism of BABA-mediated resistance against SA, we measured the activities of several well-characterized defense-related enzymes, including chitinases (CHI), β-1,3-glucanase (GLU), polyphenol oxidase (PPO), peroxidase (POX), and phenylalanine ammonia-lyase (PAL).

**Figure 4:**
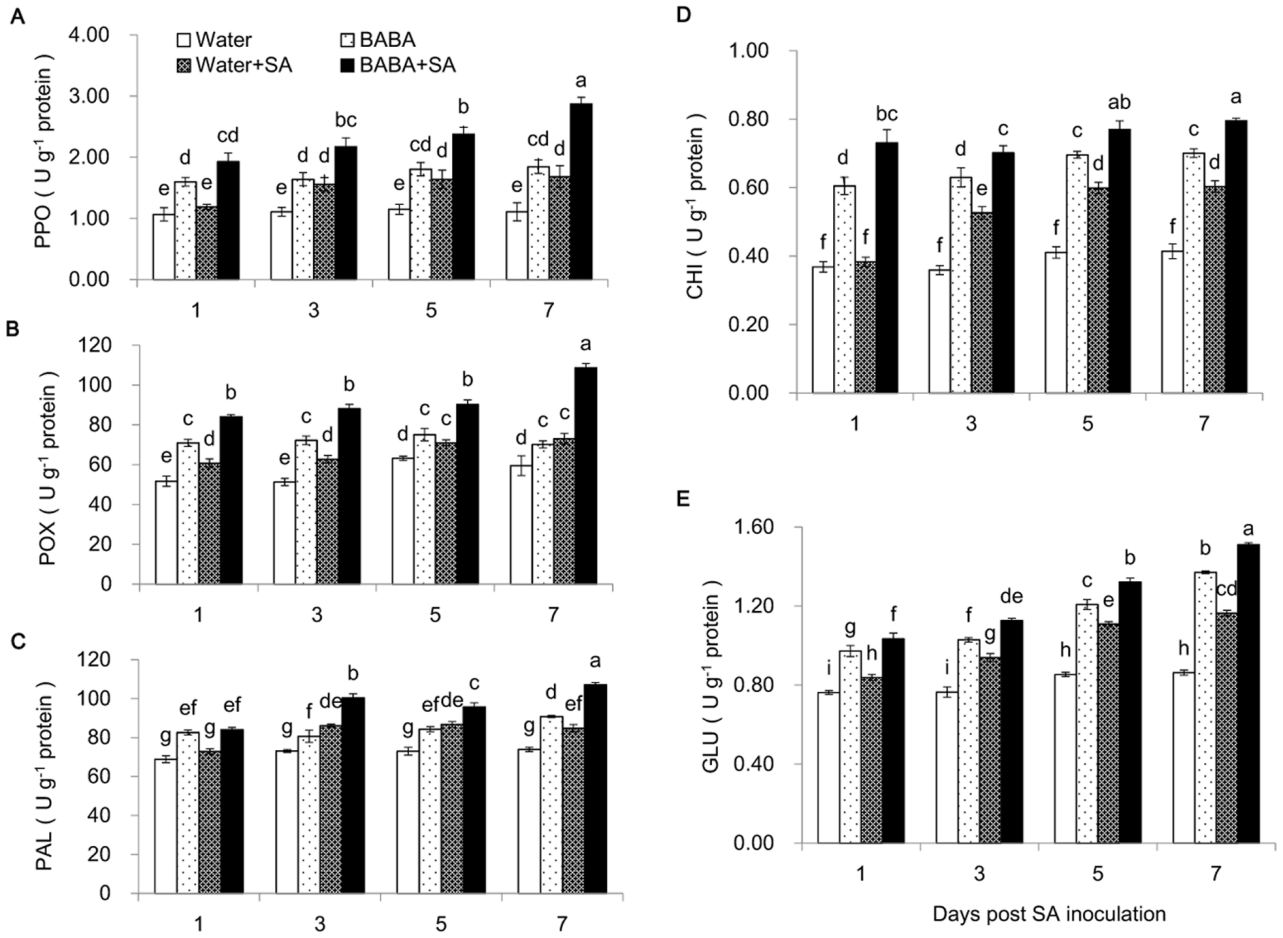
Activities of five defense related enzymes in soybean seedlings pre-treated by BABA or water post SA inoculation. (A) Polyphenol oxidase (PPO). (B) Peroxidase (POX). (C) Phenylalanine ammonia-lyase (PAL). (D) Chitinases (CHI). (E) β-1, 3-glucanase (GLU). The seedlings were pre-treated with 25 ml 25 mM BABA or water for 3 days. Bars indicate Mean±SE of three replicates. Different letters indicate significant differences between treatments according to Fisher's LSD test (P<0.05).

PPO and POX are involved in phenolic metabolism and phenolic compounds are related to plant biotic stress response. PPO that catalyzes the O_2_-dependent oxidation of phenolic compounds to form quinines has been proposed as a component in plant defense network [45]. In soybean seedlings, there was a sustained increase in the activities of both PPO and POX after SA inoculation during the entire study period. 25 mM BABA induced a higher level of the activities of PPO and POX compared to water drenched. In the plants inoculated with SA, the insect-induced enzyme activities were further elevated by BABA treatment (Figure 4A and 4B; Table S4).

PAL, a critical enzyme in phenylalanine metabolism pathway, is an indicator in plant reaction to environmental stress [46]. We found that SA inoculation increased its activity in soybean seedlings. The effect of BABA on PAL was similar to those found in the other defensive enzymes (Figure 4C; Table S4).

As the markers of systemic acquired resistance, CHI and GLU belong to the PR-2 and PR-3 families and catalyze the hydrolysis of chitin and β-1,3-glucan, respectively [47]. SA attack stimulated the activities of the two enzymes, whereas BABA treatment led to more robust increases (Figure 4D and 4E; Table S4). Consistently, augmented induction of the enzyme activities by aphid feeding were observed in soybean plants drenched with BABA compared with the control counterparts.

Taken together, these results suggested that many defensive enzymes/proteins participated in the soybean-SA interaction based on the priming triggered by BABA.

### Defense genes were upregulated in BABA-induced priming in soybean against SA attack

**Figure 5:**
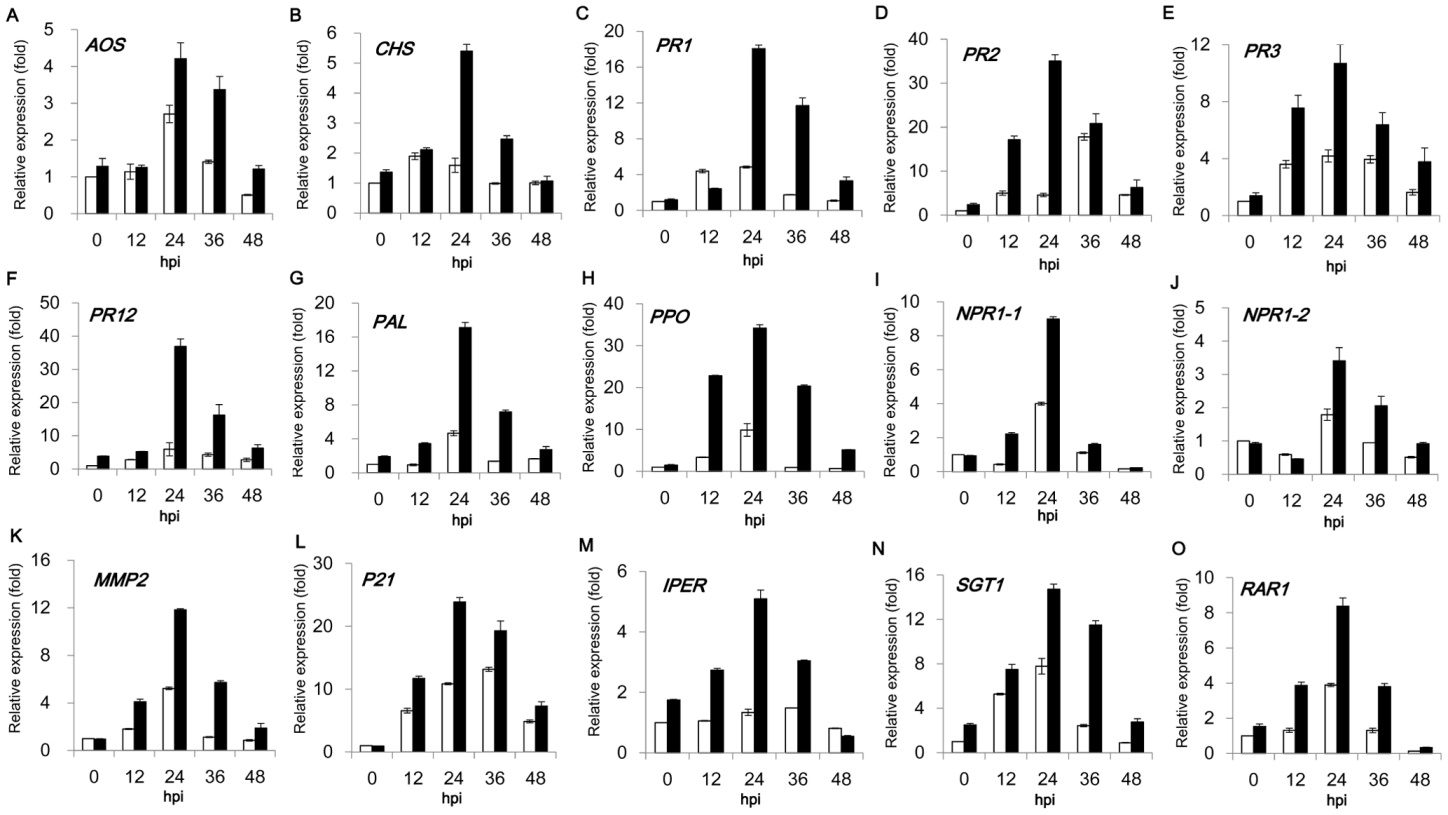
Relative expression of fifteen defense genes response to SA attack in BABA- and water-drenched soybean seedlings. (A–O) Real-time quantitative RT-PCR for fifteen genes. RNAs were extracted at designed hours post inoculation (hpi) with 6 apterous aphids. The seedlings were pre-treated with 25 ml 25 mM BABA or water for 3 days. Values were expression levels normalized to the water control. The value of seedlings drenched by water 0 h post inoculation was designed as 1. Bars indicate Mean±SE of three replicates (white bars: Water+SA; black bars: BABA+SA).

We hypothesized that BABA-enhanced plant resistance against SA was attributable to its ability to boost expression of defense-related genes. Therefore, transcript levels of 15 defense-related genes were examined in soybean seedlings after SA inoculation (Figure 5; Table S5). All of these representative genes were induced by SA attack in a similar manner with a maximum accumulation at 24 h post inoculation. Interestingly, BABA treatment was found to synergistically elevate the transcriptional induction by SA for the analyzed defense genes in almost all examined time points. For example, at the 24-h time point, transcript levels of all these genes were significantly higher in BABA-treated group than the mock control and 14 genes except *AOS* exhibited more than 2-fold induction by BABA compared to untreated seedlings (Figure 5). These results suggested that BABA could switch plants to a more alarmed state in response to SA attack.

## Discussion

### BABA-induced resistance against SA in soybean

Priming is usually defined as a sensitization to stress responsiveness. Primed plants are more resistant to biotic and abiotic stress [2], [3], [48]–[50]. High amounts of BABA inhibited height and biomass in *Arabidopsis* by restraining cell division in the meristematic tissue of the root and also reduced length of silique and seed production; nonetheless low concentration of BABA enhanced resistance of plants with little damage effect [11], [15], [51], [52]. In our study, the number of SA on BABA-treated soybean seedlings was decreased remarkably compared to control. Primed soybean seedlings inhibited the MRGR and MRRR of SA in a short time (one week). The same effect of BABA against aphid was also found in tic beans [22], and several species of Brassicaceae [17]. Similar to the long-lasting effect in tomato shown by a previous study [53], BABA still efficiently restricted the SA number in our non-choice and choice test during a longer test period (three weeks), especially in the non-choice test. Consistently, descendants of BABA-primed *Arabidopsis thaliana* plants also exhibited more resistance to the bacteria *Pseudomonas syringae* pv *tomato* (*PstavrRpt2*) and the oomycete pathogen *Hyaloperonospora arabidopsidis* without additional treatment [54].

### Induced activation of defense-related enzymes and genes

Priming is regarded as a part of systemic immunity responses in plants, but the mechanism(s) of priming are still not completely understood [55]. Based on previous studies, BABA has been known to induce a broad of defense mechanisms, depending on the type of pathogens and plants. It is capable to produce reactive oxygen species (hypersensitivity response) and enhances physical barrier by callose deposition and lignin accumulation in the cell walls [15], [56], [57]. The other mechanism mediated by BABA is through the alternation of biochemical response to the stress. For example, it promotes biosynthesis of secondary metabolites (phenols, anthocyanin and phytoalexins) and elevates activity of enzymes associated with active oxygen species, lignification and plant secondary metabolism [51], [58]–[63]. Additionally, activation of defense genes and accumulation of PR proteins involved in the antimicrobial activity were identified in many BABA-treated plants, such as tomato, peppers, potato and rape [64]–[67], though there were also contrary examples showing no accumulation of PR proteins after root application [68], [69]. In this study, some defense related enzymes were activated by BABA, such as PAL, POX and POD, which were all involved in the response to SA attack. The similar phenomenon was also reported in artichoke, cucumber, apple, grapefruit [12], [16], [70], [71]. Therefore, we believe that this is a common phenomenon in BABA-treated plants.

Priming has now been considered as a critical process in various types of systemic plant immunity, such as systemic acquired resistance (SAR) and induced systemic resistance (ISR) [55]. In our study, BABA-induced priming in soybean exhibited the canonical SAR characters. The markers of SAR, chitinase and β-1, 3-glucanase [23] were found to be activated in BABA-treated soybean seedlings and the activation of these two SAR markers reached to a higher level in the BABA-treated seedlings compared to untreated plants during the SA inoculation. Several *PR* genes (*PR1*, *PR2*, *PR3*, *PR12*, *P21-like*) also showed inducible expression in our BABA-mediated priming assay. In *Arabidopsis*, NPR1 protein is a key transcription co-activator of SAR [72] and its overexpression primed *Arabidopsis* and rice to enhance PR gene activation and immunity [73], [74]. NPR1 was also important for the primed defense response to *Hyaloperonospora arabidopsidis* during BABA-IR and the ISR response induced by *Pseudomonas fluorescens* WCS417r [75]. The consistent result was also found in soybean treated with 2, 6-dichloroisonicotinic acid (INA, another SAR inducer, functionally similar to BABA) when inoculated with *Phytophthora sojae* [42].

In addition to these defense genes and enzymes, other genes participated in resistance against SA, such as allene oxide synthase (*AOS*), matrix metalloproteinase 2 (*MMP2*) and chalcone synthase (*CHS*), were also upregulated by BABA. *AOS* is involved in oxylipin biosynthesis pathway [36]. Oxylipins are known as antibiotic factors and defense stimulating signal molecules in the plant biotic stress response [76], [77]. Accumulation of oxylipins was found both in phloem sap and aphids feeding on plants, thus oxylipins may play a direct role in plant-aphid interaction [78]. We also found the up-regulation of *AOS* by aphid feeding, suggesting its possible role in soybean against SA. Matrix metalloproteinase 2 (*MMP2*) transcript accumulated rapidly following infection with oomycete pathogen *Phytophthora sojae* or the bacterial pathogen *Pseudomonas syringae* pv. *glycinea* and was also activated in response to wounding and dehydration in soybean [39]. *MMP2* was increased in soybean leaves and seeds in response to the fungal pathogens [79]. In our study, *MMP2* was found to participate in pest defense such as SA, revealing its broad-spectrum resistance in biotic stress. *CHS* is an important gene in phenylpropanoid pathway [37] and its upregulation was accompanied by an increased flavonoid content in sunflower resistance against the downy mildew pathogen. Interestingly, two QTLs (qRa_1, LG A2 and qRa_2, LG F) were recently discovered to be linked to resistance to soybean aphid in ‘Zhongdou 27’, a cultivar with high isoflavone content, and these two QTLs were concurrently associated with high isoflavone content. Defense against SA in ‘zhongdou 27’ was likely contributed by isoflavone-mediated antibiosis process [80]. These evidences suggest that one of mechanisms underlying BABA-induced resistance against SA in soybean may be dependent on the increase of isoflavone and flavonoid content. More experimental data are needed to substantiate this hypothesis.

In our study, appropriate concentration of BABA against soybean aphids when it was applied soybean seedlings as root drenching was 25 mM in glasshouse experiments, but this concentration was still high and the efficacy may be weakened in complex field conditions. There are three methods of BABA application, root drenching, leaf spraying and seed soaking in previous study. The concentration of BABA is different among those ways. Leaf spraying may need the least concentration and researches on this domain may be valuable for its field application. In order to guarantee the induced effects of BABA, seedlings should not be watered for 2 or 3 days after BABA application, which was very important in the field. Seedlings pre-treated by BABA may resist soybean aphids by restraining their reproduction and growth rate without additional insecticide when the soybean aphid disaster was comparatively light at the initial stage. Therefore, the questions that BABA application may be conducive to reduce environmental pollution of chemical insecticide and the cost of soybean production need to be further extensive and in-depth study.

In summary, our study indicates that BABA application is a promising approach in protection soybean from aphid infestation. However, the dose of BABA should be cautiously evaluated before its practical application, since it will cause harmful consequences for soybean growth at an improper high concentration. We provided some insights into mechanisms underlying the BABA-mediated resistance against aphid in soybean. Considering the complexity of the BABA-induced priming, future studies (e.g., identification of upstream signaling component) are still needed to further illustrate the mechanisms responsible for this biological process.

## Supporting Information

Table S1 **Effects of BABA treatment on the growth of soybean seedlings.**
(DOCX)

Table S2 **Damage index (DI) of soybean seedlings treated by BABA.**
(DOCX)

Table S3 **Numbers of soybean aphids on soybean seedlings drenched with BABA and water.**
(DOCX)

Table S4 **Activities of five defense related enzymes.**
(DOCX)

Table S5 **Relative expression of fifteen defense genes response to SA attack.**
(DOCX)
