# Supplementary material for: DL-β-Aminobutyric Acid-Induced Resistance in Soybean against Aphis glycines Matsumura (Hemiptera: Aphididae)
Source: PLoS One. 2014 Jan 15;9(1):e85142. doi: 10.1371/journal.pone.0085142 (PMC3893187; doi:10.1371/journal.pone.0085142)
Supplement: Table S1 — Effects of BABA treatment on the growth of soybean seedlings. (DOCX) [file pone.0085142.s001.docx]

**Table S1. Effects of different concentrations of BABA treatment on the growth of soybean seedlings**

| **Table S1 A. Effects of different concentrations of BABA treatment on the plant height of soybean seedlings (cm) (n=20)** | | | | | | | | | | | | | | | | | | | | | | |
| --- | --- | --- | --- | --- | --- | --- | --- | --- | --- | --- | --- | --- | --- | --- | --- | --- | --- | --- | --- | --- | --- | --- |
| Concentration | Repeat | | | | | | | | | | | | | | | | | | | | Mean | SE |
| 0 | 33.6 | 25.8 | 31.4 | 30.8 | 30.7 | 30.9 | 31.2 | 30.3 | 28.5 | 32.3 | 33.5 | 32.3 | 29.5 | 28.8 | 31.4 | 32.7 | 31.5 | 29.8 | 28.5 | 33.1 | 30.83 | 0.44 |
| 10mM | 26.3 | 32.5 | 28.8 | 32.4 | 28.4 | 31.3 | 27.2 | 31.1 | 25.5 | 29.6 | 27.4 | 29.5 | 30.4 | 31.3 | 28.6 | 28.1 | 29.5 | 27.9 | 28.4 | 27.4 | 29.08 | 0.44 |
| 25mM | 31.5 | 27.2 | 27.4 | 29.7 | 29.6 | 26.7 | 25.5 | 29.8 | 24.5 | 31.6 | 27.5 | 29.5 | 27.3 | 25.8 | 28.6 | 26.3 | 25.9 | 28.5 | 26.7 | 27.9 | 27.88 | 0.44 |
| 50mM | 20.3 | 19.7 | 22.7 | 20.3 | 22.1 | 20.2 | 19.9 | 17.7 | 18.6 | 17.6 | 21.4 | 18.5 | 19.3 | 19.8 | 18.8 | 20.5 | 16.5 | 17.9 | 18.5 | 17.2 | 19.38 | 0.36 |
| 75mM | 17.3 | 20.6 | 15.2 | 17.9 | 17.3 | 18.8 | 18.6 | 19.1 | 17.6 | 19.2 | 16.2 | 18.8 | 18.2 | 19.3 | 15.3 | 15.9 | 16.3 | 18.5 | 18.9 | 19.2 | 17.91 | 0.33 |
| 100mM | 17.1 | 16.8 | 18.2 | 17.9 | 19.6 | 15.8 | 17.2 | 18.4 | 15.2 | 16.2 | 14.7 | 17.5 | 18.4 | 16.5 | 17.3 | 14.2 | 15.5 | 16.8 | 16.2 | 17.2 | 16.84 | 0.30 |

| **Table S1 B. Effects of different concentrations of BABA treatment on the fresh weight of soybean seedlings (g) (n=20)** | | | | | | | | | | | | | | | | | | | | | | |
| --- | --- | --- | --- | --- | --- | --- | --- | --- | --- | --- | --- | --- | --- | --- | --- | --- | --- | --- | --- | --- | --- | --- |
| Concentration | Repeat | | | | | | | | | | | | | | | | | | | | Mean | SE |
| 0 | 2.25 | 2.63 | 2.40 | 2.54 | 2.68 | 3.03 | 2.58 | 2.31 | 2.40 | 2.49 | 2.47 | 2.66 | 2.69 | 2.75 | 2.98 | 3.02 | 2.59 | 2.56 | 2.55 | 2.79 | 2.62 | 0.05 |
| 10mM | 2.41 | 2.44 | 2.64 | 2.38 | 2.57 | 2.68 | 2.18 | 2.39 | 2.38 | 2.19 | 2.43 | 2.54 | 2.62 | 2.90 | 2.52 | 2.40 | 2.29 | 2.81 | 2.97 | 2.85 | 2.53 | 0.05 |
| 25mM | 2.67 | 1.91 | 2.11 | 1.94 | 1.96 | 2.50 | 2.54 | 2.64 | 2.75 | 2.73 | 1.92 | 2.19 | 2.50 | 2.57 | 2.68 | 2.40 | 2.52 | 2.18 | 2.58 | 2.20 | 2.37 | 0.07 |
| 50mM | 1.23 | 1.15 | 1.16 | 1.63 | 1.40 | 1.35 | 1.11 | 1.13 | 1.32 | 0.94 | 1.09 | 1.08 | 1.08 | 1.30 | 1.01 | 1.13 | 1.09 | 1.25 | 1.06 | 1.16 | 1.18 | 0.04 |
| 75mM | 0.89 | 0.86 | 0.89 | 0.72 | 1.06 | 1.02 | 0.85 | 0.43 | 0.84 | 1.06 | 1.17 | 0.92 | 0.91 | 1.00 | 1.03 | 0.86 | 0.86 | 0.98 | 0.96 | 0.93 | 0.91 | 0.03 |
| 100mM | 0.57 | 0.64 | 0.57 | 0.31 | 0.57 | 0.68 | 0.56 | 0.68 | 0.41 | 0.74 | 0.52 | 0.40 | 0.61 | 0.46 | 0.47 | 0.60 | 0.61 | 0.54 | 0.58 | 0.46 | 0.55 | 0.02 |

| **Table S1 C. Effects of different concentrations of BABA treatment on the root fresh weight of soybean seedlings (g) (n=20)** | | | | | | | | | | | | | | | | | | | | | | |
| --- | --- | --- | --- | --- | --- | --- | --- | --- | --- | --- | --- | --- | --- | --- | --- | --- | --- | --- | --- | --- | --- | --- |
| Concentration | Repeat | | | | | | | | | | | | | | | | | | | | Mean | SE |
| 0 | 0.43 | 0.49 | 0.53 | 0.64 | 0.79 | 0.66 | 0.53 | 0.35 | 0.43 | 0.46 | 0.49 | 0.78 | 0.57 | 0.76 | 0.78 | 0.79 | 0.48 | 0.65 | 0.59 | 0.45 | 0.58 | 0.03 |
| 10mM | 0.67 | 0.51 | 0.94 | 0.72 | 0.71 | 0.50 | 0.50 | 0.26 | 0.41 | 0.43 | 0.44 | 0.74 | 0.37 | 0.56 | 0.41 | 0.47 | 0.51 | 0.61 | 0.68 | 0.58 | 0.55 | 0.04 |
| 25mM | 0.52 | 0.19 | 0.45 | 0.36 | 0.39 | 0.59 | 0.47 | 0.76 | 0.67 | 0.70 | 0.32 | 0.40 | 0.59 | 0.46 | 0.68 | 0.73 | 0.41 | 0.29 | 0.39 | 0.39 | 0.49 | 0.04 |
| 50mM | 0.22 | 0.19 | 0.18 | 0.42 | 0.18 | 0.16 | 0.20 | 0.19 | 0.22 | 0.12 | 0.18 | 0.18 | 0.16 | 0.18 | 0.19 | 0.22 | 0.21 | 0.16 | 0.17 | 0.15 | 0.19 | 0.01 |
| 75mM | 0.16 | 0.15 | 0.23 | 0.13 | 0.20 | 0.27 | 0.15 | 0.14 | 0.15 | 0.19 | 0.57 | 0.22 | 0.12 | 0.12 | 0.17 | 0.16 | 0.17 | 0.14 | 0.17 | 0.16 | 0.19 | 0.02 |
| 100mM | 0.13 | 0.13 | 0.16 | 0.08 | 0.18 | 0.19 | 0.22 | 0.16 | 0.13 | 0.10 | 0.09 | 0.14 | 0.10 | 0.12 | 0.13 | 0.18 | 0.19 | 0.15 | 0.15 | 0.13 | 0.14 | 0.01 |

| **Table S1 D. Effects of different concentrations of BABA treatment on the dry weight of soybean seedlings (g) (n=20)** | | | | | | | | | | | | | | | | | | | | | | |
| --- | --- | --- | --- | --- | --- | --- | --- | --- | --- | --- | --- | --- | --- | --- | --- | --- | --- | --- | --- | --- | --- | --- |
| Concentration | Repeat | | | | | | | | | | | | | | | | | | | | Mean | SE |
| 0 | 0.33 | 0.41 | 0.40 | 0.35 | 0.41 | 0.45 | 0.38 | 0.38 | 0.42 | 0.43 | 0.36 | 0.41 | 0.44 | 0.44 | 0.40 | 0.39 | 0.42 | 0.39 | 0.43 | 0.40 | 0.40 | 0.01 |
| 10mM | 0.32 | 0.42 | 0.43 | 0.35 | 0.46 | 0.38 | 0.31 | 0.38 | 0.42 | 0.48 | 0.45 | 0.33 | 0.38 | 0.41 | 0.40 | 0.43 | 0.35 | 0.36 | 0.37 | 0.42 | 0.39 | 0.01 |
| 25mM | 0.37 | 0.34 | 0.36 | 0.33 | 0.31 | 0.39 | 0.40 | 0.42 | 0.42 | 0.43 | 0.38 | 0.41 | 0.42 | 0.37 | 0.43 | 0.36 | 0.34 | 0.39 | 0.33 | 0.40 | 0.38 | 0.01 |
| 50mM | 0.26 | 0.25 | 0.25 | 0.26 | 0.30 | 0.27 | 0.24 | 0.27 | 0.23 | 0.27 | 0.29 | 0.27 | 0.28 | 0.26 | 0.30 | 0.26 | 0.24 | 0.29 | 0.28 | 0.28 | 0.27 | 0.00 |
| 75mM | 0.24 | 0.20 | 0.22 | 0.24 | 0.28 | 0.23 | 0.19 | 0.23 | 0.24 | 0.25 | 0.21 | 0.23 | 0.26 | 0.22 | 0.24 | 0.27 | 0.22 | 0.24 | 0.26 | 0.24 | 0.23 | 0.01 |
| 100mM | 0.29 | 0.23 | 0.20 | 0.23 | 0.26 | 0.25 | 0.18 | 0.22 | 0.25 | 0.19 | 0.20 | 0.19 | 0.22 | 0.24 | 0.26 | 0.25 | 0.20 | 0.21 | 0.27 | 0.24 | 0.23 | 0.01 |

| **Table S1 E. Effects of different concentrations of BABA treatment on the root dry weight of soybean seedlings (g) (n=20)** | | | | | | | | | | | | | | | | | | | | | | |
| --- | --- | --- | --- | --- | --- | --- | --- | --- | --- | --- | --- | --- | --- | --- | --- | --- | --- | --- | --- | --- | --- | --- |
| Concentration | Repeat | | | | | | | | | | | | | | | | | | | | Mean | SE |
| 0 | 0.06 | 0.08 | 0.11 | 0.07 | 0.10 | 0.09 | 0.08 | 0.08 | 0.09 | 0.10 | 0.06 | 0.08 | 0.08 | 0.09 | 0.11 | 0.07 | 0.08 | 0.09 | 0.09 | 0.08 | 0.08 | 0.00 |
| 10mM | 0.07 | 0.09 | 0.09 | 0.09 | 0.11 | 0.09 | 0.07 | 0.11 | 0.10 | 0.12 | 0.08 | 0.07 | 0.09 | 0.08 | 0.05 | 0.08 | 0.07 | 0.07 | 0.07 | 0.08 | 0.08 | 0.00 |
| 25mM | 0.11 | 0.06 | 0.09 | 0.08 | 0.07 | 0.09 | 0.08 | 0.10 | 0.11 | 0.10 | 0.08 | 0.06 | 0.07 | 0.06 | 0.07 | 0.01 | 0.01 | 0.08 | 0.05 | 0.06 | 0.07 | 0.01 |
| 50mM | 0.04 | 0.05 | 0.05 | 0.05 | 0.06 | 0.05 | 0.05 | 0.05 | 0.03 | 0.05 | 0.06 | 0.04 | 0.04 | 0.05 | 0.06 | 0.06 | 0.04 | 0.05 | 0.05 | 0.05 | 0.05 | 0.00 |
| 75mM | 0.04 | 0.03 | 0.02 | 0.03 | 0.03 | 0.03 | 0.02 | 0.04 | 0.03 | 0.02 | 0.02 | 0.03 | 0.04 | 0.03 | 0.02 | 0.02 | 0.03 | 0.03 | 0.04 | 0.03 | 0.03 | 0.00 |
| 100mM | 0.03 | 0.03 | 0.03 | 0.03 | 0.03 | 0.03 | 0.02 | 0.03 | 0.03 | 0.02 | 0.02 | 0.01 | 0.02 | 0.03 | 0.03 | 0.02 | 0.02 | 0.02 | 0.03 | 0.03 | 0.02 | 0.00 |

| **Table S1 F. Effects of different concentrations of BABA treatment  on the root vitality of soybean seedlings (ug g^-1^ h^-1^) (n=3)** | | | | | |
| --- | --- | --- | --- | --- | --- |
| Concentration | Repeat | | | Mean | SE |
| 0 | 93.04 | 79.29 | 85.54 | 85.96 | 3.97 |
| 10mM | 82.75 | 87.21 | 80.96 | 83.64 | 1.86 |
| 25mM | 74.70 | 83.88 | 70.31 | 76.30 | 4.00 |
| 50mM | 20.50 | 22.33 | 19.13 | 20.65 | 0.93 |
| 75mM | 15.04 | 13.04 | 14.54 | 14.21 | 0.60 |
| 100mM | 3.46 | 3.63 | 3.55 | 3.55 | 0.05 |

| **Table S1 G. Effects of different concentrations of BABA treatment on the root length of soybean seedlings (cm) (n=20)** | | | | | | | | | | | | | | | | | | | | | | |
| --- | --- | --- | --- | --- | --- | --- | --- | --- | --- | --- | --- | --- | --- | --- | --- | --- | --- | --- | --- | --- | --- | --- |
| Concentration | Repeat | | | | | | | | | | | | | | | | | | | | Mean | SE |
| 0 | 16.8 | 18.7 | 15.2 | 17.3 | 16.2 | 15.6 | 18.2 | 15.4 | 18.4 | 17.5 | 15.2 | 17.3 | 16.8 | 15.1 | 17.9 | 16.1 | 16.7 | 16.3 | 15.8 | 18.5 | 16.75 | 0.27 |
| 10mM | 17.4 | 16.8 | 19.4 | 16.9 | 15.8 | 17.3 | 18.5 | 19.2 | 18.3 | 16.3 | 17.8 | 18.6 | 17.9 | 15.3 | 18.8 | 15.6 | 16.3 | 17.2 | 17.4 | 16.2 | 17.35 | 0.27 |
| 25mM | 15.3 | 16.3 | 18.9 | 17.1 | 17.2 | 15.9 | 18.2 | 16.2 | 15.9 | 17.5 | 15.9 | 17.9 | 17.1 | 15.8 | 16.4 | 15.6 | 15.1 | 17.3 | 18.2 | 15.4 | 16.66 | 0.25 |
| 50mM | 21.5 | 19.8 | 18.5 | 20.7 | 20.8 | 18.9 | 19.5 | 20.5 | 21.1 | 19.9 | 18.9 | 18.9 | 18.3 | 19.8 | 20.5 | 21.2 | 20.9 | 19.5 | 19.7 | 21.6 | 20.03 | 0.23 |
| 75mM | 19.6 | 18.8 | 17.9 | 21.9 | 19.8 | 20.6 | 21.7 | 19.5 | 18.9 | 21.7 | 20.9 | 19.6 | 20.1 | 21.6 | 17.6 | 20.5 | 19.9 | 20.5 | 21.5 | 19.8 | 20.12 | 0.28 |
| 100mM | 19.9 | 19.5 | 20.5 | 21.5 | 18.9 | 20.4 | 17.9 | 19.3 | 19.8 | 21.8 | 18.9 | 19.5 | 20.4 | 20.6 | 18.5 | 19.5 | 19.7 | 18.9 | 20.1 | 19.9 | 19.78 | 0.21 |
