# Supplementary material for: DL-β-Aminobutyric Acid-Induced Resistance in Soybean against Aphis glycines Matsumura (Hemiptera: Aphididae)
Source: PLoS One. 2014 Jan 15;9(1):e85142. doi: 10.1371/journal.pone.0085142 (PMC3893187; doi:10.1371/journal.pone.0085142)
Supplement: Table S2 — Damage index (DI) of soybean seedlings treated by BABA. (DOCX) [file pone.0085142.s002.docx]

| **Table S2. Damage index (DI) of soybean seedlings treated by different concentrations of BABA (n=20)** | | | | | | | | | | | | | | | | | | | | | | |
| --- | --- | --- | --- | --- | --- | --- | --- | --- | --- | --- | --- | --- | --- | --- | --- | --- | --- | --- | --- | --- | --- | --- |
| Concentration | Repeat | | | | | | | | | | | | | | | | | | | | Mean | SE |
| 0 | 2 | 1 | 1 | 2 | 2 | 2 | 2 | 2 | 2 | 1 | 1 | 2 | 1 | 1 | 2 | 1 | 1 | 1 | 2 | 1 | 1.50 | 0.11 |
| 10mM | 1 | 1 | 1 | 2 | 1 | 1 | 1 | 1 | 1 | 1 | 2 | 2 | 2 | 2 | 2 | 2 | 1 | 2 | 2 | 2 | 1.50 | 0.11 |
| 25mM | 1 | 2 | 2 | 1 | 2 | 2 | 2 | 2 | 2 | 1 | 2 | 2 | 1 | 1 | 2 | 1 | 1 | 2 | 2 | 2 | 1.65 | 0.11 |
| 50mM | 3 | 3 | 4 | 3 | 2 | 4 | 4 | 3 | 2 | 4 | 3 | 4 | 3 | 4 | 4 | 3 | 4 | 3 | 4 | 3 | 3.35 | 0.15 |
| 75mM | 4 | 4 | 5 | 4 | 4 | 3 | 4 | 4 | 4 | 4 | 4 | 3 | 5 | 4 | 4 | 3 | 5 | 4 | 5 | 4 | 4.05 | 0.14 |
| 100mM | 5 | 5 | 4 | 5 | 5 | 5 | 5 | 3 | 4 | 5 | 4 | 5 | 5 | 5 | 4 | 5 | 5 | 5 | 5 | 4 | 4.65 | 0.13 |

DI scale was as follows: 1, healthy with no symptoms; 2, primary leaves yellowing with less than 50% proportion and the first trifoliolate leaf was normal; 3, primary leaves yellowing with more than 50% proportion and the size of first trifoliolate leaves was smaller; 4, primary leaves drying and the size of first trifoliolate leaves was smaller; 5, both of primary leaves and the first trifoliolate leaves drying.
