# Supplementary material for: DL-β-Aminobutyric Acid-Induced Resistance in Soybean against Aphis glycines Matsumura (Hemiptera: Aphididae)
Source: PLoS One. 2014 Jan 15;9(1):e85142. doi: 10.1371/journal.pone.0085142 (PMC3893187; doi:10.1371/journal.pone.0085142)
Supplement: Table S3 — Numbers of soybean aphids on soybean seedlings drenched with BABA and water. (DOCX) [file pone.0085142.s003.docx]

**Table S3. Numbers of soybean aphids on soybean seedlings drenched with BABA and water**

| **Table S3 A. Numbers of soybean aphids on soybean seedlings drenched with BABA or water (n=15)** | | | | | | | | | | | | | | | | | | |
| --- | --- | --- | --- | --- | --- | --- | --- | --- | --- | --- | --- | --- | --- | --- | --- | --- | --- | --- |
| Days post SA inoculation | Treatment | Repeat | | | | | | | | | | | | | | | Mean | SE |
| 1 | Water+SA | 6 | 6 | 6 | 6 | 6 | 6 | 6 | 6 | 6 | 6 | 6 | 6 | 6 | 6 | 6 | 6.00 | 0.00 |
|  | 25mM BABA+SA | 6 | 6 | 6 | 6 | 6 | 6 | 6 | 6 | 6 | 6 | 6 | 6 | 6 | 6 | 6 | 6.00 | 0.00 |
| 3 | Water+SA | 20 | 53 | 22 | 34 | 44 | 39 | 29 | 37 | 30 | 27 | 57 | 22 | 26 | 29 | 32 | 33.40 | 2.83 |
|  | 25mM BABA+SA | 6 | 7 | 16 | 19 | 11 | 21 | 12 | 14 | 9 | 11 | 13 | 18 | 24 | 8 | 15 | 13.60 | 1.36 |
| 5 | Water+SA | 102 | 52 | 67 | 78 | 82 | 89 | 59 | 62 | 72 | 57 | 79 | 91 | 73 | 69 | 58 | 72.67 | 3.69 |
|  | 25mM BABA+SA | 19 | 32 | 17 | 8 | 42 | 27 | 41 | 21 | 35 | 23 | 34 | 28 | 51 | 22 | 21 | 28.07 | 2.89 |
| 7 | Water+SA | 116 | 97 | 153 | 137 | 146 | 123 | 179 | 143 | 132 | 155 | 164 | 152 | 149 | 143 | 92 | 138.73 | 6.11 |
|  | 25mM BABA+SA | 29 | 57 | 49 | 44 | 33 | 23 | 17 | 51 | 12 | 19 | 37 | 41 | 37 | 23 | 17 | 32.60 | 3.61 |

| **Table S3 B. Numbers of soybean aphids on soybean seedlings drenched with BABA or water in choice and non-choice tests (n=15)** | | | | | | | | | | | | | | | | | | |
| --- | --- | --- | --- | --- | --- | --- | --- | --- | --- | --- | --- | --- | --- | --- | --- | --- | --- | --- |
| Days post SA inoculation | Treatment | Repeat | | | | | | | | | | | | | | | Mean | SE |
| 7 | BABA+ | 63 | 32 | 25 | 67 | 23 | 31 | 39 | 47 | 33 | 63 | 38 | 41 | 27 | 38 | 53 | 41 | 4 |
|  | Water+ | 79 | 84 | 97 | 172 | 82 | 128 | 91 | 179 | 204 | 114 | 142 | 159 | 164 | 133 | 109 | 129 | 10 |
|  | BABA | 57 | 37 | 21 | 33 | 56 | 25 | 27 | 34 | 19 | 32 | 43 | 27 | 35 | 47 | 49 | 36 | 3 |
|  | Water | 134 | 119 | 139 | 117 | 144 | 159 | 137 | 217 | 138 | 164 | 124 | 154 | 102 | 158 | 187 | 146 | 7 |
| 15 | BABA+ | 139 | 174 | 95 | 167 | 280 | 116 | 89 | 147 | 79 | 157 | 119 | 146 | 213 | 210 | 159 | 153 | 14 |
|  | Water+ | 326 | 253 | 369 | 264 | 292 | 290 | 252 | 248 | 330 | 279 | 248 | 268 | 325 | 310 | 287 | 289 | 9 |
|  | BABA | 236 | 133 | 224 | 109 | 152 | 142 | 166 | 146 | 230 | 220 | 215 | 174 | 217 | 131 | 127 | 175 | 11 |
|  | Water | 410 | 370 | 290 | 266 | 420 | 297 | 330 | 320 | 390 | 290 | 329 | 275 | 263 | 287 | 279 | 321 | 14 |
| 21 | BABA+ | 186 | 162 | 166 | 253 | 180 | 230 | 165 | 146 | 125 | 196 | 195 | 136 | 157 | 182 | 158 | 176 | 9 |
|  | Water+ | 390 | 320 | 470 | 450 | 270 | 550 | 325 | 480 | 490 | 360 | 380 | 450 | 440 | 390 | 420 | 412 | 19 |
|  | BABA | 320 | 364 | 420 | 282 | 343 | 272 | 285 | 275 | 390 | 384 | 410 | 320 | 350 | 285 | 310 | 334 | 13 |
|  | Water | 360 | 520 | 380 | 430 | 610 | 390 | 550 | 510 | 510 | 370 | 490 | 340 | 470 | 430 | 370 | 449 | 21 |

BABA+ and Water+: non-chioce test; BABA and Water: choice test.

| **Table S3 C. Numbers of soybean aphids on soybean seedlings drenched with BABA or water 7 days post SA inoculation in the second choice test (n=16)** | | | | | | | | | | | | | | | | | | |
| --- | --- | --- | --- | --- | --- | --- | --- | --- | --- | --- | --- | --- | --- | --- | --- | --- | --- | --- |
| Treatment | Repeat | | | | | | | | | | | | | | | | Mean | SE |
| Water+SA | 98 | 124 | 108 | 85 | 128 | 104 | 113 | 95 | 138 | 147 | 141 | 89 | 151 | 147 | 137 | 129 | 121 | 6 |
| 25mM BABA+SA | 31 | 41 | 21 | 38 | 19 | 26 | 26 | 18 | 28 | 34 | 24 | 33 | 22 | 19 | 42 | 52 | 30 | 2 |
