# Supplementary material for: DL-β-Aminobutyric Acid-Induced Resistance in Soybean against Aphis glycines Matsumura (Hemiptera: Aphididae)
Source: PLoS One. 2014 Jan 15;9(1):e85142. doi: 10.1371/journal.pone.0085142 (PMC3893187; doi:10.1371/journal.pone.0085142)
Supplement: Table S4 — Activities of five defense related enzymes. (DOCX) [file pone.0085142.s004.docx]

**Table S4. Activities of five defense related enzymes in soybean seedlings pre-treated by BABA or water post SA inoculation**

| **Table S4 A. PPO activity of soybean seedlings pre-treated by BABA or water post SA inoculation (U g^-1^ protein) (n=3)** | | | | | | |
| --- | --- | --- | --- | --- | --- | --- |
| Days post SA inoculation | Treatment | Repeat | | | Mean | SE |
| 1 | Water | 0.86 | 1.11 | 1.23 | 1.07 | 0.11 |
|  | BABA | 1.60 | 1.48 | 1.72 | 1.60 | 0.07 |
|  | Water+SA | 1.11 | 1.35 | 1.11 | 1.19 | 0.08 |
|  | BABA+SA | 2.22 | 1.72 | 1.85 | 1.93 | 0.15 |
| 3 | Water | 1.11 | 1.23 | 0.98 | 1.11 | 0.07 |
|  | BABA | 1.60 | 1.85 | 1.48 | 1.64 | 0.11 |
|  | Water+SA | 1.60 | 1.35 | 1.72 | 1.56 | 0.11 |
|  | BABA+SA | 2.22 | 1.97 | 2.34 | 2.17 | 0.11 |
| 5 | Water | 1.23 | 1.11 | 1.11 | 1.15 | 0.04 |
|  | BABA | 1.85 | 1.60 | 1.97 | 1.81 | 0.11 |
|  | Water+SA | 1.85 | 1.72 | 1.35 | 1.64 | 0.15 |
|  | BABA+SA | 2.34 | 2.09 | 2.71 | 2.38 | 0.18 |
| 7 | Water | 1.35 | 1.11 | 0.86 | 1.11 | 0.14 |
|  | BABA | 1.85 | 2.09 | 1.60 | 1.85 | 0.14 |
|  | Water+SA | 1.48 | 1.85 | 1.72 | 1.68 | 0.11 |
|  | BABA+SA | 2.83 | 3.08 | 2.71 | 2.87 | 0.11 |

| **Table S4 B. POX activity of soybean seedlings pre-treated by BABA or water post SA inoculation (U g^-1^ protein) (n=3)** | | | | | | |
| --- | --- | --- | --- | --- | --- | --- |
| Days post SA inoculation | Treatment | Repeat | | | Mean | SE |
| 1 | Water | 50.46 | 56.62 | 48.00 | 51.69 | 2.56 |
|  | BABA | 71.38 | 67.69 | 73.85 | 70.97 | 1.79 |
|  | Water+SA | 60.31 | 59.08 | 62.77 | 60.72 | 1.09 |
|  | BABA+SA | 75.08 | 92.31 | 84.92 | 84.10 | 4.99 |
| 3 | Water | 48.00 | 51.69 | 54.15 | 51.28 | 1.79 |
|  | BABA | 76.31 | 71.38 | 68.92 | 72.21 | 2.17 |
|  | Water+SA | 56.62 | 66.46 | 65.23 | 62.77 | 3.10 |
|  | BABA+SA | 88.62 | 91.08 | 84.92 | 88.21 | 1.79 |
| 5 | Water | 59.08 | 66.46 | 64.00 | 63.18 | 2.17 |
|  | BABA | 71.38 | 76.31 | 77.54 | 75.08 | 1.88 |
|  | Water+SA | 68.92 | 73.85 | 70.15 | 70.97 | 1.48 |
|  | BABA+SA | 84.92 | 93.54 | 92.31 | 90.26 | 2.69 |
| 7 | Water | 57.85 | 61.54 | 59.08 | 59.49 | 1.09 |
|  | BABA | 70.15 | 66.46 | 73.85 | 70.15 | 2.13 |
|  | Water+SA | 71.38 | 70.15 | 77.54 | 73.03 | 2.28 |
|  | BABA+SA | 112.00 | 104.62 | 109.54 | 108.72 | 2.17 |

| **Table S4 C. PAL activity of soybean seedlings pre-treated by BABA or water post SA inoculation (U g^-1^ protein) (n=3)** | | | | | | |
| --- | --- | --- | --- | --- | --- | --- |
| Days post SA inoculation | Treatment | Repeat | | | Mean | SE |
| 1 | Water | 69.42 | 65.48 | 71.63 | 68.84 | 1.80 |
|  | BABA | 81.35 | 83.69 | 82.95 | 82.67 | 0.69 |
|  | Water+SA | 69.54 | 72.86 | 76.43 | 72.94 | 1.99 |
|  | BABA+SA | 81.97 | 85.78 | 84.68 | 84.14 | 1.13 |
| 3 | Water | 70.89 | 73.23 | 75.32 | 73.15 | 1.28 |
|  | BABA | 74.58 | 82.83 | 84.68 | 80.70 | 3.10 |
|  | Water+SA | 85.29 | 84.43 | 88.74 | 86.15 | 1.32 |
|  | BABA+SA | 100.68 | 99.45 | 101.29 | 100.47 | 0.54 |
| 5 | Water | 75.45 | 70.77 | 72.86 | 73.03 | 1.35 |
|  | BABA | 84.68 | 82.83 | 85.42 | 84.31 | 0.77 |
|  | Water+SA | 84.31 | 89.35 | 86.65 | 86.77 | 1.46 |
|  | BABA+SA | 98.95 | 95.75 | 92.43 | 95.71 | 1.88 |
| 7 | Water | 75.20 | 74.83 | 71.75 | 73.93 | 1.09 |
|  | BABA | 87.51 | 90.58 | 94.40 | 90.83 | 1.99 |
|  | Water+SA | 88.86 | 83.94 | 81.72 | 84.84 | 2.11 |
|  | BABA+SA | 109.42 | 106.22 | 105.72 | 107.12 | 1.16 |

| **Table S4 D. CHI activity of soybean seedlings pre-treated by BABA or water post SA inoculation (U g^-1^ protein) (n=3)** | | | | | | |
| --- | --- | --- | --- | --- | --- | --- |
| Days post SA inoculation | Treatment | Repeat | | | Mean | SE |
| 1 | Water | 0.39 | 0.37 | 0.34 | 0.37 | 0.02 |
|  | BABA | 0.60 | 0.63 | 0.58 | 0.61 | 0.01 |
|  | Water+SA | 0.41 | 0.36 | 0.38 | 0.38 | 0.02 |
|  | BABA+SA | 0.74 | 0.69 | 0.76 | 0.73 | 0.02 |
| 3 | Water | 0.36 | 0.32 | 0.40 | 0.36 | 0.03 |
|  | BABA | 0.69 | 0.61 | 0.60 | 0.63 | 0.03 |
|  | Water+SA | 0.54 | 0.52 | 0.51 | 0.53 | 0.01 |
|  | BABA+SA | 0.68 | 0.72 | 0.70 | 0.70 | 0.01 |
| 5 | Water | 0.38 | 0.43 | 0.41 | 0.41 | 0.01 |
|  | BABA | 0.71 | 0.66 | 0.72 | 0.70 | 0.02 |
|  | Water+SA | 0.57 | 0.63 | 0.60 | 0.60 | 0.02 |
|  | BABA+SA | 0.74 | 0.78 | 0.79 | 0.77 | 0.02 |
| 7 | Water | 0.49 | 0.36 | 0.40 | 0.41 | 0.04 |
|  | BABA | 0.66 | 0.73 | 0.71 | 0.70 | 0.02 |
|  | Water+SA | 0.63 | 0.62 | 0.55 | 0.60 | 0.03 |
|  | BABA+SA | 0.80 | 0.81 | 0.78 | 0.80 | 0.01 |

| **Table S4 E. GLU activity of soybean seedlings pre-treated by BABA or water post SA inoculation (U g^-1^ protein) (n=3)** | | | | | | |
| --- | --- | --- | --- | --- | --- | --- |
| Days post SA inoculation | Treatment | Repeat | | | Mean | SE |
| 1 | Water | 0.75 | 0.76 | 0.78 | 0.76 | 0.01 |
|  | BABA | 1.01 | 0.92 | 0.99 | 0.97 | 0.03 |
|  | Water+SA | 0.86 | 0.84 | 0.82 | 0.84 | 0.01 |
|  | BABA+SA | 1.03 | 1.01 | 1.06 | 1.03 | 0.01 |
| 3 | Water | 0.78 | 0.81 | 0.71 | 0.76 | 0.03 |
|  | BABA | 1.01 | 1.02 | 1.05 | 1.03 | 0.01 |
|  | Water+SA | 0.89 | 0.98 | 0.94 | 0.94 | 0.03 |
|  | BABA+SA | 1.12 | 1.14 | 1.13 | 1.13 | 0.01 |
| 5 | Water | 0.85 | 0.88 | 0.83 | 0.85 | 0.02 |
|  | BABA | 1.19 | 1.25 | 1.18 | 1.21 | 0.02 |
|  | Water+SA | 1.11 | 1.08 | 1.13 | 1.11 | 0.01 |
|  | BABA+SA | 1.35 | 1.30 | 1.32 | 1.32 | 0.01 |
| 7 | Water | 0.81 | 0.87 | 0.91 | 0.86 | 0.03 |
|  | BABA | 1.35 | 1.37 | 1.39 | 1.37 | 0.01 |
|  | Water+SA | 1.13 | 1.17 | 1.20 | 1.16 | 0.02 |
|  | BABA+SA | 1.49 | 1.52 | 1.53 | 1.51 | 0.01 |
