# Supplementary material for: DL-β-Aminobutyric Acid-Induced Resistance in Soybean against Aphis glycines Matsumura (Hemiptera: Aphididae)
Source: PLoS One. 2014 Jan 15;9(1):e85142. doi: 10.1371/journal.pone.0085142 (PMC3893187; doi:10.1371/journal.pone.0085142)
Supplement: Table S5 — Relative expression of fifteen defense genes response to SA attack. (DOCX) [file pone.0085142.s005.docx]

**Table S5. Relative expression of fifteen defense genes response to SA attack in BABA- and water-drenched soybean seedlings**

| **Table S5 A. Relative expression (fold) of *AOS* response to SA attack post inoculation (h) (n=3)** | | | | | |  | **Table S5 B. Relative expression (fold) of *CHS* response to SA attack post inoculation (h) (n=3)** | | | | | |  | **Table S5 C. Relative expression (fold) of *PR1* response to SA attack post inoculation (h) (n=3)** | | | | | |
| --- | --- | --- | --- | --- | --- | --- | --- | --- | --- | --- | --- | --- | --- | --- | --- | --- | --- | --- | --- |
| Treatment | 0 | 12 | 24 | 36 | 48 |  | Treatment | 0 | 12 | 24 | 36 | 48 |  | Treatment | 0 | 12 | 24 | 36 | 48 |
| Water+SA | 1.00 | 1.06 | 2.23 | 1.32 | 0.48 |  | Water+SA | 1.00 | 1.82 | 1.49 | 0.99 | 0.90 |  | Water+SA | 1.00 | 4.31 | 5.62 | 1.75 | 1.13 |
|  | 1.00 | 1.21 | 2.93 | 1.46 | 0.51 |  |  | 1.00 | 2.11 | 1.82 | 0.94 | 1.09 |  |  | 1.00 | 4.11 | 4.34 | 1.70 | 1.21 |
|  | 1.00 | 1.13 | 2.96 | 1.45 | 0.53 |  |  | 1.00 | 1.75 | 1.46 | 1.02 | 1.03 |  |  | 1.00 | 4.76 | 4.58 | 1.78 | 0.90 |
| BABA+SA | 1.48 | 1.14 | 3.35 | 3.13 | 2.38 |  | BABA+SA | 1.33 | 2.05 | 4.96 | 2.30 | 1.25 |  | BABA+SA | 1.27 | 2.50 | 18.60 | 9.16 | 2.86 |
|  | 1.17 | 1.34 | 4.53 | 4.07 | 3.89 |  |  | 1.52 | 2.24 | 5.67 | 2.67 | 1.02 |  |  | 1.07 | 2.46 | 17.28 | 12.20 | 3.01 |
|  | 1.21 | 1.29 | 4.74 | 2.92 | 3.10 |  |  | 1.24 | 2.03 | 5.57 | 2.43 | 0.93 |  |  | 1.18 | 2.33 | 18.32 | 14.57 | 4.08 |

| **Table S5 D. Relative expression (fold) of *PR2* response to SA attack post inoculation (h) (n=3)** | | | | | |  | **Table S5 E. Relative expression (fold) of *PR3* response to SA attack post inoculation (h) (n=3)** | | | | | |  | **Table S5 F. Relative expression (fold) of *PR12* response to SA attack post inoculation (h) (n=3)** | | | | | |
| --- | --- | --- | --- | --- | --- | --- | --- | --- | --- | --- | --- | --- | --- | --- | --- | --- | --- | --- | --- |
| Treatment | 0 | 12 | 24 | 36 | 48 |  | Treatment | 0 | 12 | 24 | 36 | 48 |  | Treatment | 0 | 12 | 24 | 36 | 48 |
| Water+SA | 1.00 | 4.72 | 3.82 | 16.30 | 4.43 |  | Water+SA | 1.00 | 4.07 | 4.94 | 3.51 | 1.76 |  | Water+SA | 1.00 | 2.83 | 6.46 | 3.96 | 3.23 |
|  | 1.00 | 5.92 | 4.98 | 18.66 | 4.76 |  |  | 1.00 | 3.58 | 3.89 | 4.25 | 1.60 |  |  | 1.00 | 2.88 | 5.98 | 5.21 | 2.61 |
|  | 1.00 | 4.39 | 4.96 | 18.39 | 4.63 |  |  | 1.00 | 3.37 | 3.71 | 4.04 | 1.54 |  |  | 1.00 | 2.61 | 5.43 | 3.73 | 2.42 |
| BABA+SA | 2.59 | 15.61 | 36.86 | 19.31 | 6.39 |  | BABA+SA | 1.39 | 7.58 | 11.98 | 7.19 | 3.26 |  | BABA+SA | 4.02 | 5.30 | 40.72 | 14.93 | 7.04 |
|  | 2.48 | 18.43 | 33.49 | 22.32 | 6.58 |  |  | 1.28 | 6.90 | 9.71 | 6.45 | 3.87 |  |  | 3.71 | 5.17 | 37.12 | 17.61 | 6.34 |
|  | 2.12 | 17.41 | 34.96 | 20.94 | 5.89 |  |  | 1.49 | 8.12 | 10.35 | 5.49 | 4.19 |  |  | 3.84 | 5.30 | 32.87 | 16.05 | 5.46 |

| **Table S5 G. Relative expression (fold) of *PAL* response to SA attack post inoculation (h) (n=3)** | | | | | |  | **Table S5 H. Relative expression (fold) of *PPO* response to SA attack post inoculation (h) (n=3)** | | | | | |  | **Table S5 I. Relative expression (fold) of *NPR1-1* response to SA attack post inoculation (h) (n=3)** | | | | | |
| --- | --- | --- | --- | --- | --- | --- | --- | --- | --- | --- | --- | --- | --- | --- | --- | --- | --- | --- | --- |
| Treatment | 0 | 12 | 24 | 36 | 48 |  | Treatment | 0 | 12 | 24 | 36 | 48 |  | Treatment | 0 | 12 | 24 | 36 | 48 |
| Water+SA | 1.00 | 1.15 | 5.02 | 1.42 | 1.71 |  | Water+SA | 1.00 | 3.51 | 9.07 | 1.03 | 0.75 |  | Water+SA | 1.00 | 0.40 | 3.86 | 1.08 | 0.17 |
|  | 1.00 | 0.81 | 4.72 | 1.33 | 1.61 |  |  | 1.00 | 3.18 | 9.86 | 0.89 | 0.70 |  |  | 1.00 | 0.39 | 3.81 | 1.05 | 0.17 |
|  | 1.00 | 0.86 | 4.26 | 1.36 | 1.65 |  |  | 1.00 | 3.36 | 10.72 | 0.89 | 0.68 |  |  | 1.00 | 0.50 | 4.35 | 1.23 | 0.18 |
| BABA+SA | 2.01 | 3.63 | 18.26 | 7.27 | 3.11 |  | BABA+SA | 1.28 | 25.05 | 35.35 | 20.80 | 6.19 |  | BABA+SA | 0.86 | 2.16 | 8.86 | 1.56 | 0.22 |
|  | 1.99 | 3.38 | 16.30 | 6.76 | 2.90 |  |  | 1.74 | 22.21 | 34.58 | 19.72 | 4.84 |  |  | 0.96 | 2.11 | 8.85 | 1.54 | 0.24 |
|  | 1.73 | 3.33 | 16.79 | 7.51 | 2.34 |  |  | 1.45 | 21.20 | 32.63 | 20.46 | 4.41 |  |  | 0.97 | 2.38 | 9.25 | 1.72 | 0.23 |

| **Table S5 J. Relative expression (fold) of *NPR1-2* response to SA attack post inoculation (h) (n=3)** | | | | | |  | **Table S5 K. Relative expression (fold) of *MMP2* response to SA attack post inoculation (h) (n=3)** | | | | | |  | **Table S5 L. Relative expression (fold) of *P21* response to SA attack post inoculation (h) (n=3)** | | | | | |
| --- | --- | --- | --- | --- | --- | --- | --- | --- | --- | --- | --- | --- | --- | --- | --- | --- | --- | --- | --- |
| Treatment | 0 | 12 | 24 | 36 | 48 |  | Treatment | 0 | 12 | 24 | 36 | 48 |  | Treatment | 0 | 12 | 24 | 36 | 48 |
| Water+SA | 1.00 | 0.57 | 1.77 | 0.95 | 0.52 |  | Water+SA | 1.00 | 1.74 | 4.95 | 1.05 | 0.73 |  | Water+SA | 1.00 | 7.31 | 10.68 | 13.81 | 5.16 |
|  | 1.00 | 0.58 | 1.60 | 0.94 | 0.48 |  |  | 1.00 | 1.79 | 5.31 | 1.19 | 0.88 |  |  | 1.00 | 6.34 | 10.82 | 12.71 | 5.03 |
|  | 1.00 | 0.64 | 1.99 | 0.95 | 0.55 |  |  | 1.00 | 1.88 | 5.41 | 1.12 | 0.95 |  |  | 1.00 | 6.09 | 11.04 | 12.97 | 4.40 |
| BABA+SA | 0.92 | 0.45 | 3.25 | 1.72 | 0.88 |  | BABA+SA | 1.03 | 3.68 | 11.93 | 5.50 | 2.26 |  | BABA+SA | 0.88 | 12.39 | 25.73 | 16.78 | 8.15 |
|  | 0.85 | 0.44 | 2.81 | 1.84 | 0.88 |  |  | 0.88 | 4.22 | 11.66 | 5.65 | 1.78 |  |  | 0.92 | 11.23 | 21.70 | 19.48 | 7.60 |
|  | 0.99 | 0.48 | 4.16 | 2.62 | 1.00 |  |  | 0.93 | 4.40 | 11.93 | 6.00 | 1.61 |  |  | 1.00 | 11.50 | 24.22 | 21.60 | 6.13 |

| **Table S5 M. Relative expression (fold) of *IPER* response to SA attack post inoculation (h) (n=3)** | | | | | |  | **Table S5 N. Relative expression (fold) of *SGT1* response to SA attack post inoculation (h) (n=3)** | | | | | |  | **Table S5 O. Relative expression (fold) of *RAR1* response to SA attack post inoculation (h) (n=3)** | | | | | |
| --- | --- | --- | --- | --- | --- | --- | --- | --- | --- | --- | --- | --- | --- | --- | --- | --- | --- | --- | --- |
| Treatment | 0 | 12 | 24 | 36 | 48 |  | Treatment | 0 | 12 | 24 | 36 | 48 |  | Treatment | 0 | 12 | 24 | 36 | 48 |
| Water+SA | 1.00 | 1.03 | 1.33 | 1.49 | 0.79 |  | Water+SA | 1.00 | 5.34 | 9.10 | 2.65 | 0.94 |  | Water+SA | 1.00 | 1.08 | 3.87 | 1.15 | 0.12 |
|  | 1.00 | 1.08 | 1.34 | 1.49 | 0.80 |  |  | 1.00 | 5.12 | 7.55 | 2.37 | 0.85 |  |  | 1.00 | 1.39 | 3.44 | 1.21 | 0.12 |
|  | 1.00 | 1.07 | 1.35 | 1.48 | 0.84 |  |  | 1.00 | 5.38 | 6.70 | 2.31 | 0.90 |  |  | 1.00 | 1.48 | 4.38 | 1.57 | 0.14 |
| BABA+SA | 1.79 | 2.77 | 4.54 | 3.10 | 0.48 |  | BABA+SA | 2.78 | 7.15 | 15.64 | 12.70 | 3.28 |  | BABA+SA | 1.39 | 3.76 | 8.33 | 3.64 | 0.33 |
|  | 1.74 | 2.63 | 5.25 | 3.04 | 0.55 |  |  | 2.30 | 8.37 | 14.23 | 10.31 | 2.25 |  |  | 1.40 | 3.62 | 7.59 | 3.64 | 0.30 |
|  | 1.73 | 2.81 | 5.50 | 3.00 | 0.59 |  |  | 2.43 | 6.98 | 14.30 | 11.48 | 2.78 |  |  | 1.82 | 4.24 | 9.22 | 4.15 | 0.38 |
